# Effect of forced swimming stress on *in-vivo* fertilization capacity of rat and subsequent offspring quality

**DOI:** 10.4103/0974-1208.63120

**Published:** 2010

**Authors:** Ghasem Saki, Fakher Rahim, Ozra Allah Vaysi

**Affiliations:** Department of Anatomical Sciences, Ahwaz; 1 Laboratory of Cell Culture, Faculty of Medicine, Ahwaz; 2 Physiology Research Center, Ahwaz Joundishapour University, Ahwaz; 3 Apadana Clinical Research Center, Private Hospital of Apadana, Ahwaz

**Keywords:** Fertilization capacity, *in vivo* fertilization, rat, stress

## Abstract

**AIMS:**

This study aimed to determine the effect of 50 days of forced swimming stress on fertilization capacity of rat and subsequent offspring quality.

**SETTING AND DESIGN:**

The prospective study designed *in vivo*.

**MATERIALS AND METHODS:**

Total 90 Wistar rats including 30 adult male (3 months of age, weighing 210 ± 10.6 g) and 60 female rats (3 months of age, weighing 230 ± 12.2 g) were engaged in this study. Male rats were randomly divided in two equal groups (*n* = 15): Control and experimental groups. Animals of the experimental group were submitted to forced swimming stress for 3 min in water at 32°C daily for 50 days. Then all adult male rats were mated with normal females (2 per each male) for 7 days. Female rats were sacrificed and autopsy was performed on day 20 of pregnancy when uterus and ovaries were examined for the number of corpora lutea, dead and live fetuses, embryo resorption, implantation sites, and fetus weight.

**CONCLUSION:**

Results of this study have important implications for families attempting pregnancy. Stress pursuant to life events may have a negative impact on *in vivo* fertilization capacity of male rats and subsequent offspring quality.

## INTRODUCTION

Infertility is the inability of a couple to become pregnant after unprotected sexual intercourse (after 1 year). In the United States, about 10% of couples are affected by infertility.[1] According to the American Society for Reproductive Medicine, around 33% of the diagnosis is due to female infertility, 33% of the time it is linked to male infertility, and the rest 33% is due to a combination of factors from both partners.[1] The cause of the couple infertility cannot be determined in roughly 20% of the cases.[1] Psychological stress has been compromised as one of the major causes of idiopathic infertility in both men and women.[2–7]

Many studies have investigated that psychological causes affect male factor infertility,[8–14] but it remains difficult to tease out stress as a cause or consequence of infertility. A diversity of stress factors such as micro-organisms, hyperthermia, and exposure to heavy metals inhibit male reproductive functions and spermatogenesis.[15] After implementation of the stressful stimuli such as prolonged immobilization and forced swimming stress, similar effects were observed.[16,17] The previous study demonstrated that forced swimming stress in the time course equal spermatogenesis period, i.e., 48-50 days,[17] in the rat will be significantly effective to reduce the number and motility of sperm as well as the *in vitro* fertilization capacity.[18] The purpose of the present study was to determine whether forced swimming stress applied to adult male rat may affect *in vivo* fertilization capacity and subsequent offspring quality.

## MATERIALS AND METHODS

This study was conducted from March 2009 to August 2009. A total 30 adult male Wistar rats, 3 months of age, weighing 210 ± 10.6 g were purchased from Laboratory Animals Care and Breeding Centre of Ahwaz Jondishapour University of Medical Sciences, Ahwaz, Iran. The fertilizing ability of male mice was proven at the beginning of the experiment by selecting post-first-wave of spermatogenesis that mate and observed positive pregnancy.[19] All rats were randomly divided into two equal groups (*n* = 15): Control and experimental groups. All animals were housed individually per cage under a 12-h light/dark cycle, 20 ± 2°C temperature and 60-65% humidity-controlled room with food and water ad libitum. All procedures were approved by international guidelines and by the Institute Research Ethics and Animal Care and Use Committee of Ahwaz Jondishapour University of Medical Sciences.

### Experimental design

As the control group remained in their cages, the experimental group was submitted to forced swimming for 3 min in water at 32°C daily for 50 days. Stress was sized up by the hot-plate test after the last stressing session.[17] In the hot-plate, the plate temperature was 52°C and maximal cut-off time was 60 seconds. The latency time for the hind licking after exposure to the hot-plate surface was measured and the increase in relation to control was considered to be an index of the anti-nociceptive effect.

### Fertility assessment

Adult control and stressed males were mated with sexually mature normal females presenting at least three regular cycles confirmed by the analysis of daily vaginal smears. Females in the *pro-oestrus* stage in the morning were mated with male rats overnight (*two females per one male*). The presence of spermatozoa in the vaginal smear on the next morning was indicative of copulation and was considered as day-zero of pregnancy. Autopsy was performed on day 20 of pregnancy when uterus and ovaries were examined for the number of corpora lutea, dead and live fetuses, and embryo resorption and implantation sites (total amount of live and dead fetuses plus embryo resorption). The rates of preimplantation loss (corpora lutea minus implantation sites) and postimplantation loss (implantation sites minus live fetuses) were then determined.[20]

### Statistical analysis

Data are stated as a mean ± standard deviation (SD) and percentage. The statistical significance of difference between the control and experimental groups was determined by using the Chi-square. Differences between the mean were considered to be significant when *P* < 0.05 was achieved.

## RESULTS

In the hot plate, 3 min swims at 32°C significantly prolonged (*P* < 0.05) the latency time for the hind paws latency (15.3 ± 1.66 s and 28.7 ± 2.33 s) for control and stressed rats [Table 1]. The pregnancy rate in female that mated with control and stressed male was 83% (25/30) and 60% (18/30 females), respectively. Statistical analysis showed significant differences between two groups (*P* < 0.05). The numbers of live fetuses (as estimate male fertility rate) in female group that mated with control and stressed males were 6.4 ± 0.5 and 5 ± 0.2 per uterus, respectively. The numbers of live fetuses per uterus showed significant difference between two groups (*P* < 0.05). There was no difference (*P* > 0.05) in weight of live fetuses between two groups of study [Table 1]. Implantation sites were observed in 170 and 125 points of uteruses of pregnant female that mated with nonstress and stressed male rats, respectively. These values showed significant difference between two female groups (*P* < 0.05). The rate of pre- and postimplantation loss in female mated with stressed male rats increased significantly (*P* < 0.05).

**Table 1:** Effect of 50 days of forced swimming stress on fertilization capacity of rat and subsequent offspring quality

Variable	Control rats (*n* = 15)	Forced swimming stressed rats (*n* = 15)
		
Experimental groups				
Latency time for the hind paws licking (second)	15.3 ± 1.66	28.7 ± 2.33
Number of female rats who mate with male rats	30	30
Pregnant females (%)	25 (83)	18 (60)
Corpora lutea	179	131
Live fetuses (Mean ± SD)	161 (6.4 ± 0.5 per uterus)	90 (5 ± 0.2 per uterus)
Body weight (gram)	5.6 ± 2.2	5.3 ± 1.7
Dead fetuses	2	5
Resorption sites	7	30
Implantation sites	170	125
Preimplantation loss	9	6
Postimplantation loss	9	35

## DISCUSSION

It is clear from the above observation that the forced swimming stress in rat will be effective to reduce *in vivo* fertilization capacity. The reduced fertilization capacity of stressed male is probably due to decrease in semen quality such as count, motility, and morphology. The association between psychosocial stress and decreased semen quality has biologic plausibility supported by numerous studies. Most hypothesized pathways by which stress may impact semen quality operate under endocrine factors. In animals, induced psychological stress negatively affects testosterone, LH levels, sexual behavior, and semen parameters.[21] It was previously reported that immobilization stress reduced the size and weight of testis as well as marked suppression of spermatogenesis and also suppressed all stages of cell division and maturity of spermatogenesis.[22] Moreover, in this study we observed that the rate of pre- and postimplantation loss in female rats mated with stressed male rats significantly increased. Almeida and coworker demonstrated that adult animals submitted to intermittent immobilization from prepuberty presented a significant decrease in fertility rates, confirmed by a more than twofold increase in both pre- and postimplantation loss in the progeny of normal females.[16] The cause of this manifestation is probably due to fertilization of oocytes with damaged spermatozoa. There are some reports about the deleterious effect of stress on animal and human semen quality such as a reduction in seminal volume, sperm concentration and motility, and in the amount of normal spermatozoa.[23–25] These manifestations are less obvious in men, being generally associated with extreme physical or psychological situation and are considered to be the basis of temporary infertility.[22]

## CONCLUSION

Present study demonstrates that forced swimming after the period of time necessary to complete an entire cycle of the spermatogenesis, between 48 and 53 days, when applied to adult male rats, the number of sperm, fertilization capacity, as well as motility of sperm will decrease significantly.
